# Ameloblastoma of the Maxilla With Distant Metastases to the Lungs: A Case Report and Literature Review

**DOI:** 10.7759/cureus.63233

**Published:** 2024-06-26

**Authors:** Hadi Afandi Al-Hakami, Baraa I Awad, Renad M Alsolamy, Mohammed Al-Garni, Mohammad A Alshareef, Murad Essatari

**Affiliations:** 1 Otolaryngology: Head and Neck Surgical Oncology and Reconstructive Surgery, King Saud bin Abdulaziz University for Health Sciences, King Abdullah International Medical Research Center, Ministry of the National Guard - Health Affairs, Jeddah, SAU; 2 College of Medicine, Otolaryngology-Head & Neck Surgery, King Saud bin Abdulaziz University for Health Sciences, King Abdullah International Medical Research Center, Ministry of the National Guard - Health Affairs, Jeddah, SAU; 3 College of Medicine, King Saud bin Abdulaziz University for Health Sciences, King Abdullah International Medical Research Center, Jeddah, SAU; 4 College of Medicine, Otolaryngology - Head & Neck Surgery, King Saud bin Abdulaziz University for Health Sciences, King Abdullah International Medical Research Center, Ministry of the National Guard - Health Affairs, Jeddah, SAU; 5 Otolaryngology - Head and Neck Surgery, King Abdulaziz Medical City, Jeddah, SAU; 6 Pathology and Laboratory Medicine, King Abdul-Aziz Medical City, Western Region (WR) National Guard Health Affairs, King Abdullah International Medical Research Center, Ministry of the National Guard - Health Affairs, Jeddah, SAU

**Keywords:** pulmonary metastasis, recurrence, malignant ameloblastoma, odontogenic tumour, ameloblastoma

## Abstract

Maxillary ameloblastoma is one of the rarest odontogenic epithelial tumors encountered, as 80% of ameloblastomas are seen within the mandible. Ameloblastoma is usually incidentally detected in the third to fourth decades of life, as most patients remain asymptomatic; yet some patients may complain of a slowly growing, painless swelling.

We present a case of maxillary ameloblastoma with pulmonary metastasis along with a brief literature review. A 17-year-old male initially presented with painless right facial swelling, which, on examination, was non-tender, immobile, irregular, pink in color, with a high tendency to bleed, and located in the mucogingival sulcus with a size of around 3x2.5 cm. Following comprehensive radiological and histopathological evaluation, the diagnosis of ameloblastoma characterized by the coexistence of plexiform and follicular patterns was confirmed. The patient underwent a partial right maxillectomy with an obturator sealing the hard palate. Unfortunately, multiple local recurrences were identified afterward, and eventually, pulmonary metastasis was detected.

Early and adequate surgical resection of the primary tumor is crucial to prevent further recurrences in patients with ameloblastoma. This could be achieved by providing a tight postoperative follow-up schedule while paying special attention to the lungs, neck, and other suspicious areas to detect metastasis as early as possible.

## Introduction

Ameloblastomas are rare, locally aggressive odontogenic epithelial tumors that account for 1% of jaw tumors [1-2]. The mandible is involved in up to 80% of the cases, whereas the maxilla is affected in the remaining 20% [3]. Ameloblastoma is usually detected incidentally in the third to fourth decades of life, as most patients remain asymptomatic, although some patients may complain of slowly growing, painless swelling along with pain and paresthesia, albeit less commonly [3-4]. Radiological investigations, such as CT, can provide insight into the extent of the tumor; however, the mainstay of an ameloblastoma diagnosis is biopsy and histological examination where follicular and plexiform patterns are mostly identified [3,5].

The differential diagnosis of malignant ameloblastoma involves distinguishing it from other odontogenic and non-odontogenic neoplasms due to its rarity and aggressive nature. Key considerations include differentiating it from ameloblastic carcinoma, primary intraosseous carcinoma, and metastatic carcinoma to the jaw [6]. Clinical, radiographic, and histopathological evaluations are crucial. Malignant ameloblastomas often present with similar histological features to benign ameloblastomas but exhibit malignant behavior, such as distant metastases, most commonly to the lungs [7]. Immunohistochemical staining and molecular analysis can aid in differentiation, with markers such as Ki-67 and p53 providing insights into proliferative and tumor suppressor activity, respectively [7].

Ameloblastomas are generally classified into three main types: solid multi-cystic, unicystic, and peripheral, with different management approaches depending on the extent of invasion. In fact, solid multi-cystic ameloblastomas are considered locally invasive and resistant to conservative therapies. On the other hand, both unicystic and peripheral types are less aggressive and can respond well to conservative management [1].

Overall, the management of ameloblastoma depends on eradicating the affected tissue, along with clean reconstruction of the remaining parts, which makes it a challenging goal to achieve. This highlights the importance of individualized surgical planning and treatment based on both patient factors and tumor factors [1,3]. Ameloblastoma is still a subject of concern and controversy because of its convoluted behavior. Here, we report a case of maxillary ameloblastoma with pulmonary metastasis. To our knowledge, this is the first report of pulmonary metastasis in Saudi Arabia.

## Case presentation

2006

A 17-year-old male patient was referred to the Ear, Nose, and Throat Department at National Guard Hospital, Jeddah, Saudi Arabia, in 2006, complaining of a painless right facial swelling that had lasted for two months. The swelling was gradually increasing in size, and there were no reports of weight loss, loss of appetite, or difficulty in breathing or swallowing. On examination, the patient was in good general condition. The observed swelling exhibited characteristics of being non-tender, immobile, irregular in shape, pink in color, with a propensity for bleeding, and situated within the buccogingival sulcus in close association with the right maxillary molar teeth No. 17 and 18. Its dimensions were approximately 3 x 2.5 cm. The ears, nose, and throat (ENT) examination was unremarkable. There was no lymph node involvement. A biopsy from the right gingival mass suggested the presence of islands or nests of odontogenic epithelium within a fibrous stroma and anastomosing cords or sheets within a less cellular, loosely collagenous stroma. This confirmed the presence of ameloblastoma with both plexiform and follicular histopathology. The computed tomography (CT) scan showed a soft tissue mass destroying the posterior part of the right maxilla and extending into the maxillary antrum, completely filling it, with no lymph node involvement. As a result, the patient underwent partial right maxillectomy with obturator sealing to the hard palate and received antibiotics.

2008

After two years, in 2008, the patient complained of painless swelling of the right maxilla. The swelling was compressing the eye globe, leading to reduced visual acuity. Upon evaluation, a smooth-surfaced, non-tender cystic mass was identified above the right lower orbital rim, with normal extra-ocular muscle movements. The CT scan showed a mucocele and surgical excision was performed. Histopathology results showed recurrent ameloblastoma, characterized by features such as peripheral palisading columnar cells, reverse polarity, central stellate reticulum-like cells, cystic degeneration, and occasional squamous metaplasia within the tumor islands. Postoperatively, the patient was doing well with no active complaints such as nasal obstruction, rhinorrhea, or allergic symptoms. ENT examination revealed hypertrophied inferior turbinate with pale mucosa. The patient was discharged and followed up regularly.

2011-2017

**Figure 1:**
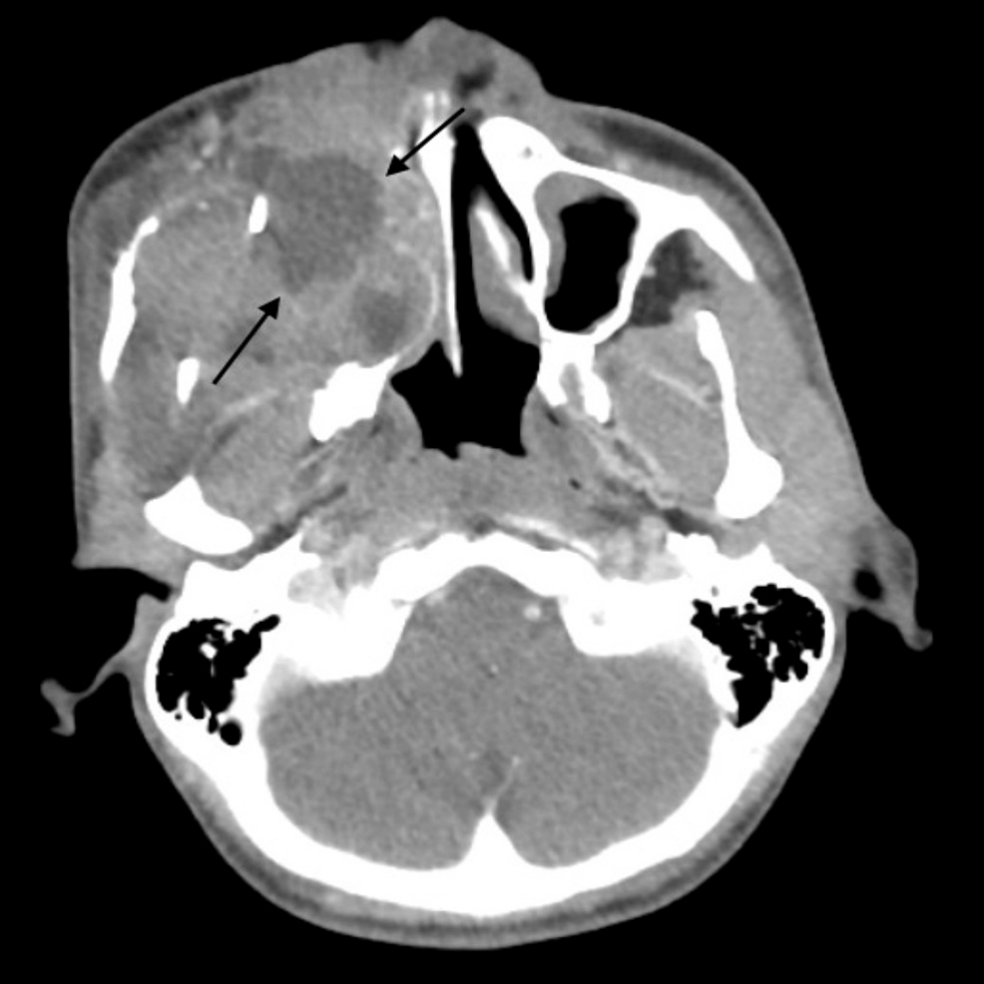
CT of paranasal sinuses taken in 2011 A large heterogeneously enhancing mass with solid and cystic components is seen in the area of the previously resected right maxillary sinus tumor measuring around 6.5 x 6.0 cm in its maximum dimensions. It erodes the adjacent bones. The lesion extends to the right nasal cavity and right ethmoidal air cells. Displaced surgical clips are seen in its anterior aspects The rest of the paranasal sinuses show partial mucosal thickening. Preservation of the fat planes between this lesion and the right orbital cavity is seen.

**Figure 2:**
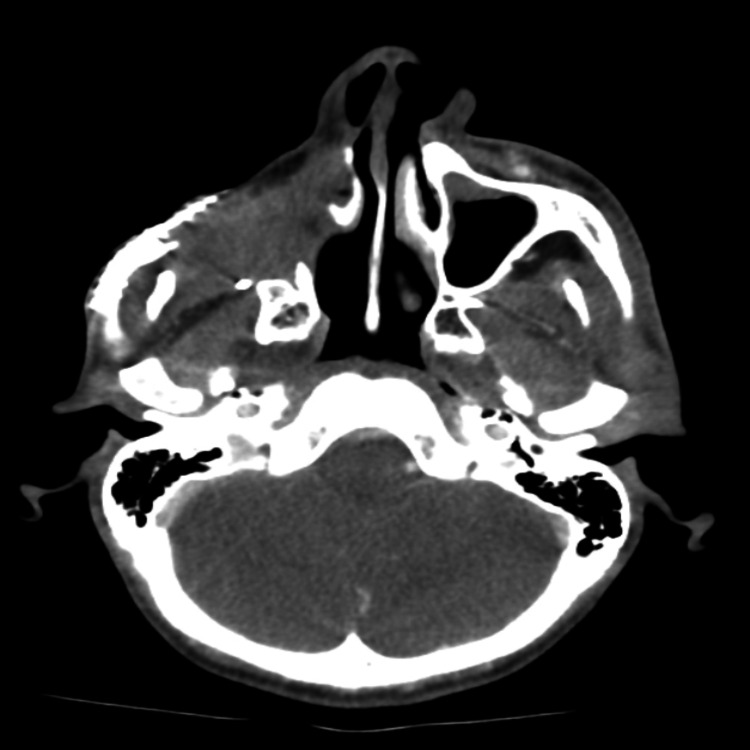
Facial bone and neck CT taken in 2017 Incidental finding of two suspicious pulmonary nodules highly concerning for metastasis, for further evaluation with a CT scan of the chest. The enlarged left side cervical lymph node needs further correlation. No discrete masses are present to suggest local recurrence at the surgical bed.

**Figure 3:**
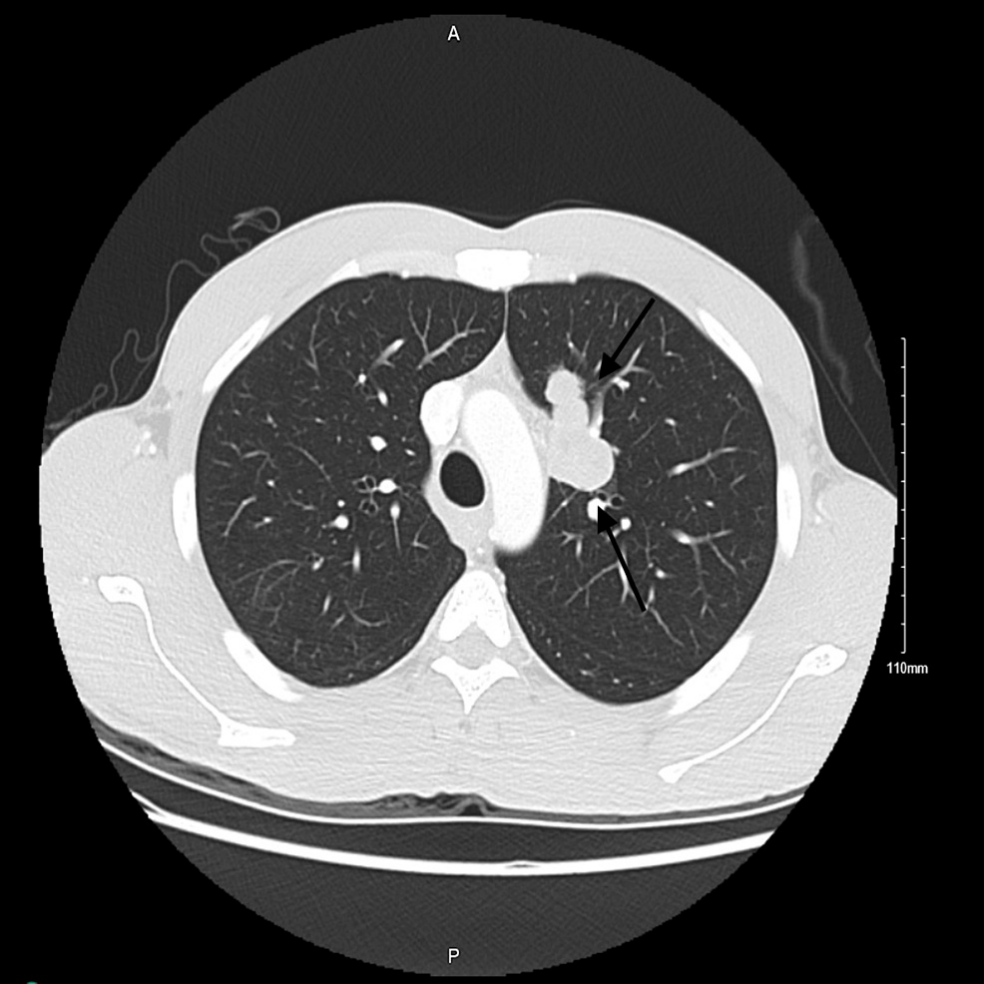
CT chest taken in 2017 There is a lobulated, left, para mediastinal, hypodense, enhancing lesion measuring 2.5 x 4.3 cm in keeping with matted metastatic nodules. There are other scattered pulmonary nodules seen in the right upper lobe, one of them showing a cavitation. A smaller cavitary pulmonary nodule is seen in the left lower lobe. No pleural effusion or pneumothorax is seen.

**Figure 4:**
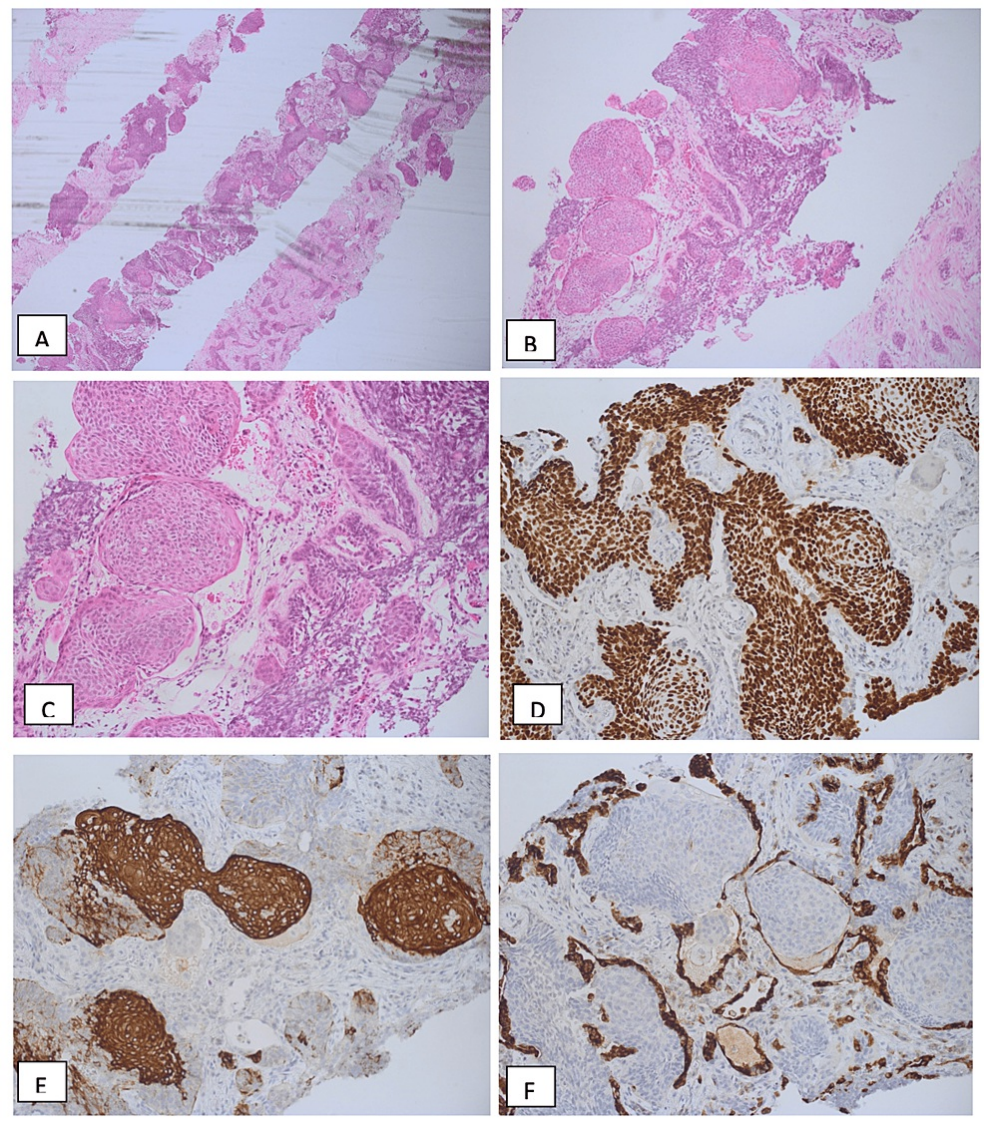
Histopathology examination with hematoxylin and eosin (H&E) and immunohistochemistry stains revealed A, B, & C: Fibrous tissue infiltrated by nests and islands of epithelial cells that exhibit peripheral palisading and reverse polarity with bland-looking nuclear morphology. No mitosis or necrosis seen. H&E, 40x, 100x, and 200x), D: P63 strong nuclear staining (IHC, 200x), E: CK 5/6 strong diffuse staining (IHC, 200x), F: epithelial antigen membrane (EMA), focally positive

In 2011, the patient presented with a recurrence of ameloblastoma, which was evidenced by a swelling in the right maxilla persisting for a duration of eight months. There was no reported history of nasal obstruction, visual impairments, feeding difficulties, trismus, or chronic ailments. Upon physical examination, the patient appeared to be in overall good health, with a noticeable mass in the right cheek region. The ear, nose, and throat (ENT) assessment revealed no significant abnormalities. Following a thorough evaluation that included a CT of the nose and paranasal sinuses (as depicted in Figure 1) and a brain MRI, a right maxillectomy was performed. This surgical procedure aimed to remove the mass while ensuring the preservation of the eye. The imaging studies revealed the presence of a sizable mass with both solid and cystic components. Moreover, the mass was located in the area of a previously excised tumor in the right maxillary sinus, measuring 6.5 x 6.0 cm at its largest dimensions. This lesion extended to the right nasal cavity and right ethmoidal air cells. Right neck dissection followed by reconstruction of the maxilla, hard palate, and orbital floor with a rectus abdominis free myocutaneous flap along with an iliac crest bone graft was performed. The patient was followed regularly up to 2017 when he presented with ectropion of the right lower eyelid and exposed plate. A facial bone and neck CT (Figure 2) excluded local recurrence and most importantly revealed incidental findings of two suspicious pulmonary nodules highly concerning for metastasis. Therefore, a CT chest (Figure 3) was ordered and showed a left lobulated para mediastinal hypodense enhancing lesion measuring 2.5 x 4.3 cm and other scattered pulmonary nodules in the right upper lobe. The patient underwent a transthoracic left lung lesion biopsy and histopathology (Figure 4) confirmed the presence of metastatic ameloblastoma. The microscopic examination showed multiple cores of fibrous tissue with no lung parenchyma. The cores were infiltrated by neoplastic growth in the form of nests and islands with prominent peripheral palisading and reverse polarity. The cells have abundant eosinophilic cytoplasm with bland-looking nuclei. No aberrant mitosis or necrosis was identified. A panel of immunohistochemistry stains was performed, including P63, CK5/6, epithelial antigen membrane (EMA), Calretinin, and CD56. The target cell was strongly positive for P63 and CK5/6 and focally for EMA but negative for Calretinin and CD56. These microscopic and immunohistochemistry findings, in addition to the clinical history, were consistent with metastatic ameloblastoma. Over the subsequent two-year period, the metastatic disease exhibited progression, characterized by the emergence of new lung lesions and the involvement of the pleural, pericardial, peritoneal, and suprahepatic regions of the inferior vena cava. Regrettably, the histological subtype demonstrated resistance to conventional chemotherapy, and the thoracic lesions were deemed inoperable by the cardiothoracic surgeon. The Tc-99m bone scan identified a potential new periodontal lesion in the left anterior-lateral aspect of the mandible. Concurrently, as the disease advanced, the patient experienced worsening dyspnea and orthopnea attributed to massive pleural effusion and an incidental pulmonary embolism. Acute kidney injury and hypercalcemia resolved within two days after pleural tapping.

## Discussion

Maxillary ameloblastoma is a rare form of odontogenic epithelial tumor, with 80% of ameloblastomas occurring in the mandible. We reviewed similar case reports from the literature. According to these reports, this subtype typically presents as a painless mass, likely due to the anatomical location of the maxilla. The tumor's proximity to the nasal cavity, nasopharynx, paranasal sinuses, orbits, and skull base, combined with the absence of a cortical plate, often results in delayed diagnosis [8]. Most cases in the literature, including ours, show painless swelling as the primary symptom. However, Len et al. reported a case of a 30-year-old male who experienced painful swelling in the right zygomatico-maxillary region, leading to a diagnosis of ameloblastoma [9].

In addition, the extensive blood supply to the area can dramatically increase the risk of invasion and spread by providing an alternative route [10]. Also, having multiple local recurrences, inadequate surgical management, and a plexiform pattern, as seen in our case, increase the rate of metastasis. The incidence of metastasizing ameloblastoma is around 2%, but more realistically, it is far less. Ameloblastoma is most likely to metastasize to the lung as seen in our case, followed by cervical lymph nodes, brain, and bone [11]. In contrast to our study, ameloblastoma had metastasized to the pelvis in a study done by Zarbo et al. where they presented a case of a 14-year-old female who was diagnosed with a spindle cell ameloblastoma and managed conservatively by curettage and excision [12]. Moreover, a 29-year-old male who was diagnosed with a plexiform pattern ameloblastoma had metastasis to the right temporal area of the brain in a case report by Rotellini et al. [13].

Table 1 presents the results of our literature review.

**Table 1:** Results of literature search for maxillary ameloblastoma

Age/sex	Presentation	Histological type	Management	Recurrence	Metastasis	References
40 years old /M	Two bony hard lumps in the left maxillary alveolar bone	Not mentioned	Extended resection of left maxilla	Yes	Yes (lungs)	[9]
46 years old /F	Not mentioned	Not mentioned	Extended resection of the right maxilla.	Yes	Yes (lungs)	[9]
30 years old /M	Painful swelling in the right zygomaticomaxillary region	Well-differentiated follicular ameloblastoma	Total enucleation and curettage	Yes	Yes (lungs)	[11]
14 years old /F	Not mentioned	Spindle cell ameloblastoma	Conservatively by curettage and excision	Yes	Yes (pelvis)	[12]
29 years old /M	Swelling of the oral cavity without any symptoms	Plexiform pattern	Extended right maxillectomy with resection of a portion of the hard palate	Yes	Yes (right temporal area of the brain)	[13]
63 years old /F	Painless and progressive swelling of the right maxilla present for two months	Basal cell type ameloblastoma	Partial left maxillectomy	No	No	[14]
16 years old /M	Painless swelling on the right cheek	Unicystic ameloblastoma mural form	Surgical enucleation of the lesion and extraction of lateral incisor tooth	No	No	[15]
56 years old /M	Swelling in the left maxillary palatal region for approximately 2 years and a gradual loosening of left maxillary central and lateral incisors, and left maxillary canine	Follicular, plexiform, and desmoplastic patterns	Left hemi maxillectomy for definitive treatment	No	Yes (lungs)	[16]
17 years old/M	Painless right facial swelling, which on examination, was non-tender, immobile, irregular, pink in color with a high tendency to bleed, and located in the buccogingival sulcus with a size of around 3x2.5 cm	Plexiform and follicular patterns	Partial right maxillectomy with obturator sealing to the hard palate	Yes	Yes (lungs)	This paper

The management of ameloblastoma is tailored based on both patient comorbidities and the type of the tumor. Several authors suggest conservative management when dealing with unicystic ameloblastoma. Enucleation, curettage, or marsupialization are the main methods of conservative therapy. Although these techniques are characterized by low morbidity and satisfactory results in terms of function and aesthetics, they are found to have a high recurrence rate (60-80%), which may limit their use [1,14]. This was demonstrated in both the Lin and Zarbo et al. studies in which a conservative method was chosen to be the initial method of treatment, but multiple recurrences followed by metastases to the lung and pelvis were observed in both cases, respectively [15-16].

Limitations

The main limitation encountered in this paper was the retrieval of medical reports and imaging for the patient. This challenge arose because the patient presented before the establishment of the electronic file system for medical records (Best Care). Additionally, the patient was originally referred to our hospital, which may have further contributed to the lack of available data.

## Conclusions

Early and adequate surgical resection of the primary tumor is crucial to prevent further recurrence in patients with ameloblastoma. Furthermore, identifying patient and tumor-related factors that can influence the rate and likelihood of recurrence is highly encouraged. In addition, ameloblastoma patients should have a tight postoperative follow-up schedule while paying special attention to the lungs, neck, and other suspicious areas to detect metastasis as early as possible.
